# A database of water chemistry in eastern Siberian rivers

**DOI:** 10.1038/s41597-022-01844-y

**Published:** 2022-11-30

**Authors:** Shiqi Liu, Ping Wang, Qiwei Huang, Olga I. Gabysheva, Zehong Li, Jialing Zhang, Ekaterina S. Kazak, Yu Liu, Tcogto Zh. Bazarzhapov, Raisa N. Shpakova, Viktor A. Gabyshev, Sergey P. Pozdniakov, Natalia L. Frolova

**Affiliations:** 1 Key Laboratory of Water Cycle and Related Land Surface Processes, Institute of Geographic Sciences and Natural Resources Research, Chinese Academy of Sciences, 11 A, Datun Road, Chaoyang District, Beijing, 100101 China; 2 University of Chinese Academy of Sciences, Beijing, 100049 China; 3 Institute for Biological Problems of Cryolithozone, Siberian Branch, Russian Academy of Sciences, Yakutsk, 677980 Russia; 4 Department of Hydrogeology, Lomonosov Moscow State University, GSP-1, Leninskie Gory, Moscow, 119899 Russia; 5 School of Environmental Science and Engineering, Guangzhou University, Guangzhou, 510006 China; 6 Baikal Institute of Nature Management of Siberian Branch of the Russian Academy of Sciences, 670047 Ulan-Ude, Russia; 7 Regional Governance and National Policy Department, Moscow State Institute of International Relations, 76, Prospect Vernadskogo, Moscow, 119454 Russia; 8 Department of Land Hydrology, Lomonosov Moscow State University, GSP-1, Leninskie Gory, Moscow, 119991 Russia

**Keywords:** Element cycles, Hydrology, Hydrology, Environmental health, Geochemistry

## Abstract

Permafrost degradation leads to considerable changes in river ecosystems. The Eastern Siberian River Chemistry (*ESRC*) database was constructed to create a spatially extensive river chemistry database to assess climate warming-induced changes in freshwater systems in permafrost-dominated eastern Siberia. The database includes 9487 major ion (Na^+^, K^+^, Ca^2+^, Mg^2+^, Cl^−^, SO_4_^2−^ and HCO_3_^−^) data of chemical results from 1434 water samples collected mainly in six large river basins in eastern Siberia spanning 1940–2019. Data were obtained from public databases, scientific literature in English and Russian, and researchers and were formatted with a consistent table structure. The database is transparent and reproducible. Climate variable (air temperature and precipitation) data, discharge data, trace element concentration data, and isotope data at the basin and subbasin scales are also provided. This database enhances knowledge about the water chemistry of the permafrost region, especially in eastern Siberia, where data are scarce. The database will be useful to those assessing spatiotemporal changes in river water chemistry associated with permafrost degradation or other environmental stressors in a warmer climate.

## Abstract (summary)

**Table:** 

Measurement(s)	water chemistry
Technology Type(s)	chromatography and spectrometry
Sample Characteristic - Environment	hydrochemistry
Sample Characteristic - Location	Eastern Siberia

## Background & Summary

The Arctic Ocean accounts for only 1% of the global ocean volume, while it receives more than 10% of global river discharge (~ 4300 km^3^ per year)^1,2^ from ~ 15% of the global land surface^3^. Surface water from Arctic and sub-Arctic river basins is generally fresh^4^ with low concentrations of dissolved ions. Over the past several decades, the Arctic freshwater system has experienced significant changes^5^ due to accelerated climate warming and an intensified hydrological cycle as well as human activities across the terrestrial pan-Arctic^6–8^.

The chemical compositions of river water are the result of natural processes and anthropogenic influences^9^. Progressive increases in major ion delivery to the Arctic and sub-Arctic freshwater systems are highly associated with permafrost degradation in a warmer climate^10^. Permafrost degradation enhances infiltration, increases groundwater storage, and drives deeper flow paths^11^, leading to increasing contributions of highly mineralized groundwater to streamflow. As a result, Arctic freshwater is shifting from a mineral-poor surface water-dominated river system to a mineral-rich groundwater system^12^. Our understanding of the response of the Arctic freshwater system to permafrost degradation is mainly based on river water chemistry observations in western Siberia^13^.

The water chemistry database in western Siberia is relatively rich, especially for the Ob River, with sampling dating back to the 1930s^13^, and is constantly replenished^14–16^. In contrast, water chemistry data in eastern Siberia are relatively sparse. Early data on water chemistry in eastern Siberia were published mainly in the Russian literature and were difficult to access. In fact, the water chemistry of eastern Siberia was continuously observed and studied by scholars in the former Soviet Union during the 1940s and 1950s e.g., Bochkarev^17^. In the 1990s, research for two PhD theses was conducted to systematically study the water chemistry of the Lena River^18^ and the other rives of eastern Siberia^19^. After 2000, the Arctic Great Rivers Observatory (ArcticGRO), which originated from the Pan-Arctic River Transport of Nutrients, Organic Matter, and Suspended Sediments (PARTNERS) project, provides open-access water chemistry data of the Lena and Kolyma Rivers from 2003. However, water chemistry data for other rivers (e.g., Angara, Selenga, Yana and Indigirka) are still limited.

**Fig. 1:**
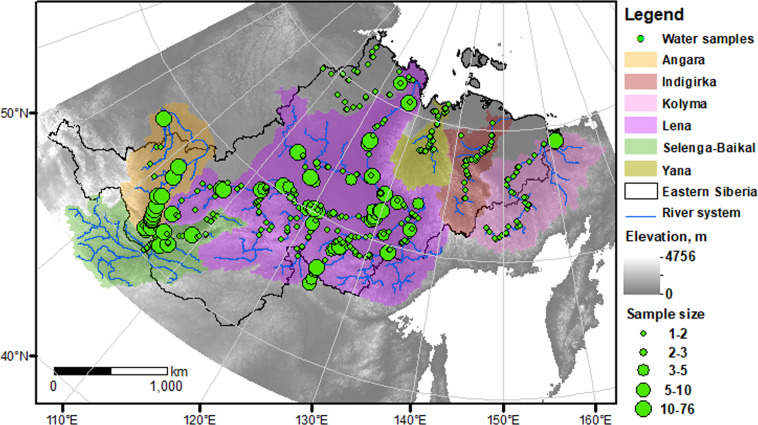
Map showing the water sample locations for the Eastern Siberian River Chemistry (*ESRC*) database. The green dots represent the sampling location, and the 5 different sizes of dots represent the sample amount; the six coloured sections represent different river basins; the black line corresponds to the eastern Siberia boundary; the grey gradient represents the elevation change, and the blue line shows the river system of each river basin.

The objective of this study was to combine existing eastern Siberian river chemistry datasets into a single database that can help assess climate effects on freshwater chemistry in permafrost-dominated regions. Data obtained from public databases, researchers, and the literature, including English and Russian articles and dissertations, were combined to create a georeferenced database with 9487 water chemistry results for 1434 samples collected from rivers across eastern Siberia (Fig. 1). A shapefile that delineated polygons for river basins was constructed to accompany the chemistry database. This database also included climate variables such as air temperature and precipitation at the basin scale. The database is transparent and reproducible and can be useful to assess the responses of freshwater systems to climate change in permafrost-dominated regions.

## Methods

### Data acquisition

Google Scholar, Scopus, and eLIBRARY.RU, as well as public data sources, were searched using the term “water chemistry” in Eastern Siberia. In total, 1434 multisource data, including major ions, were obtained from both published datasets and unpublished field studies (Table 1). Among these data, (1) 159 datasets were from the ArcticGRO water quality data^20^ and the GLObal RIver CHemistry (GLORICH) databases^21^; (2) 928 water chemistry data were sourced from 10 published studies in both English^22–26^ and Russian^17,18,27–29^); and (3) 347 unpublished datasets were provided by Gabysheva O.I. and Wang P. Chemical analyses of the waters sampled by research groups led by Gabysheva O.I. and Wang P. were performed at the laboratory of the Institute for Biological Problems of Cryolithozone and the Baikal Institute of Nature Management (Siberian Branch, Russian Academy of Sciences), respectively, following the methodology described by Semenov^30^.

**Table 1:** Data sources of the ESRC Dataset.

No.	Type	Source	*n*	River basin (region)	Period
1	Database	ArcticGRO^20^	151	Lena/Kolyma	2003–2006
					2009–2019
2	Database	GLORICH^21^	8	Lena/Yana/Indigirka	1991
					1995–1997
3	Literature	Georgiadi, *et al*.^22^	2	Lena	2010
					2018
4	Literature	Huh, *et al*.^24^	63	Lena/small rivers from eastern Siberia	1991–1997
5	Literature	Kuzmin, *et al*.^23^	3	Angara	1950–1955
					1970–1984
					1997–2007
6	Literature	Huh and Edmond^25^	51	Angara/Selenga-Baikal/Lena	1991
					1993–1994
					1996–1997
7	Literature	Huh, *et al*.^26^	80	Lena/Yana/Indigirka/Kolyma/other small rivers from eastern Siberia	1991–1992
					1995–1997
8	Literature	Berkin, *et al*.^27^	6	Angara/Selenga-Baikal	2001
9	Literature	Bochkarev^17^	391	Angara/Selenga-Baikal/Lena	Before 1955
10	Literature	Grebenshchikova, *et al*.^28^	22	Angara	Before 1955
					1957–1961
					1984–1995
					1997–2009
11	Literature	Sidorov^29^	7	Lena	1985–1990
12	Literature	Shpakova^18^	303	Lena	1993
13	Unpublished data	Gabysheva O.I.	303	Lena/Yana/Kolyma/Indigirka/other small rivers from eastern Siberia	2006–2011
14	Unpublished data	Wang P.	44	Angara/Selenga-Baikal	2015–2018

Note: (1) ArcticGRO - Arctic Great Rivers Observatory water quality dataset (https://www.arcticgreatrivers.org/data)^20^; (2) GLORICH - GLObal RIver CHemistry database (10.1594/PANGAEA.902360)^21^; (3) the database eliminates duplicate data from different sources; (4) *n* represents the number of samples.

For the 347 unpublished datasets, water samples were collected in pre-cleaned polypropylene bottles and immediately filtered through disposable sterile Sartorius filter elements (pore size 0.45 μm). The first 50 mL of the filtrate was discarded. The filtered solutions for cation and trace element analysis were acidified (pH = 2) with ultrapure double-distilled HNO_3_, stored in HDPE bottles prewashed with 1 M HCl and rinsed with Milli-Q deionized water. Filtered water samples for anions were not acidified and stored in High Density Polyethylene (HDPE) bottles prewashed according to the procedure described above for cations. Some components were analysed directly at the sampling sites; the remaining samples were fixed according to the analysis procedure and transported in a refrigerated box at 1–3 °C. Anions (Cl^−^, SO_4_^2−^, HCO_3_^−^) were determined by high-performance liquid chromatography (HPLC), and cations (Ca^2+^, Mg^2+^, K^+^ and Na^+^) were analysed by flame atomic-absorption spectrometry.

We consolidated all collected data for major dissolved ions (Na^+^, K^+^, Ca^2+^, Mg^2+^, Cl^−^, SO_4_^2−^ and HCO_3_^−^) in eastern Siberian rivers and divided them into 7 categories according to the spatial distribution in six major river basins and out-of-basin areas (named Angara, Selenga-Baikal, Lena, Yana, Indigirka, Kolyma and Eastern Siberia in the “Basin” attribute) and eliminated duplicate data.

### Unit conversion

All of the original water chemical data included major ions (Na^+^, K^+^, Ca^2+^, Mg^2+^, Cl^−^, SO_4_^2−^ and HCO_3_^−^) without alteration other than standardization of units to mg/L. Based on Lesch^31^ and EWT Water Technology (https://www.ewt-wasser.de/en/knowledge/concentration-quantities-unit-conversions.html#Umre%204/5), the atomic weight (*AW*) and valence (*V*) were used in the conversion relationships between ppm, mmol/L, mEq/L and mg/L (Table 2):

$$Concentration\;in\;[ppm]=Concentration\;in\;[mg/L]$$

$$Concentration\;in\;[mmol/L]\times AW=Concentration\;in\;[mg/L]$$

$$Concentration\;in\;[mEq/L]\times AW/V=Concentration\;in\;[mg/L]$$

**Table 2:** Unit conversion for each ionic component in the ESRC dataset.

Unit	Na^+^	K^+^	Ca^2+^	Mg^2+^	Cl^−^	SO_4_^2−^	HCO_3_^−^
mmol/L	1.0	1.0	1.0	1.0	1.0	1.0	1.0
mEq/L	1.0	1.0	2.0	2.0	1.0	2.0	1.0
ppm	22.990	39.100	40.080	24.310	35.453	96.056	61.008
mg/L	22.990	39.100	40.080	24.310	35.453	96.056	61.008

The inorganic total dissolved solids (*TDS*) were determined by the sum of seven major ions (Na^+^, K^+^, Ca^2+^, Mg^2+^, Cl^−^, SO_4_^2−^ and HCO_3_^−^) expressed in mg/L. Among the ArcticGRO datasets^20^, the SO_4_^2−^ concentrations (117 datasets) were obtained by multiplying the concentration of sulfur (mg S/L) by three, and the HCO_3_^−^ concentrations (147 datasets) were calculated from the alkalinity based on the ratio of equivalent weights^32^ and marked as “cal_alk” in the attribute “Note”:

$$Concentration\;S{O}_{4}^{2-}[mg/L]=3.0\times Concentration\;S\;[mg/L]$$

$$Concentration\;HC{O}_{3}^{-}[mg/L]=1.22\times Concentration\;CaC{O}_{3}\;[mg/L],{\rm{pH}} < 8.4$$

Anion HCO_3_^−^ in 151 groups of data from Huh, *et al*.^24^, Huh, *et al*.^26^, and GLORICH^21^ were determined by the charge balance method from the other ions, which was marked as “cal_ib” in the “Note” attribute.

### Ionic charge balance controls

To control the data quality of water samples, the ionic charge balance technique was used in this study since the concentrations of all negatively charged ions should be equal to the sum of the positively charged ions in each sample. The ion balance (*IB*) was determined as follows WMO^33^:

$$\begin{array}{lll}IS & = & \sum _{cations}{C}_{i}+\sum _{anions}{C}_{i}\\ ID & = & \sum _{cations}{C}_{i}-\sum _{anions}{C}_{i}\\ IB & = & \left(\frac{ID}{IS}\right)\times 100\end{array}$$

where *C*_*i*_ is the concentration of ion type *i* in a specific sample (mEq/L); *IS* is the sum of all ion concentrations (mEq/L); *ID* is the difference between the sum of the cation concentrations and the sum of the anion concentrations (mEq/L); and *IB* is the ratio of *ID* to *IS*, representing both systematic and random errors during the measurements.

As a result, 122 samples (8.5% of the total samples) with absolute values of *IB* greater than 10 were excluded from this study, and in 48 samples, some ions were absent (marked as “imbalance” and “absent” in the “IB” attribute, respectively). As a result, 1264 samples were considered reasonable for further analysis.

### Normal distribution assessment

The normality assumption is assessed using skewness and kurtosis and applies to both small and large samples^34^ for the 1264 sets of *TDS* data. The skewness (γ1) and kurtosis (γ2) describe the degree of asymmetry in a distribution and the extent to which the density of observations differs from the probability density of the normal curve^35^:

$$\begin{array}{lll}\gamma 1 & = & \frac{1}{n-1}\mathop{\sum }\limits_{i=1}^{n}{\left({x}_{i}-\bar{x}\right)}^{3}/S{D}^{3}\\ \gamma 2 & = & \frac{1}{n-1}\mathop{\sum }\limits_{i=1}^{n}{\left({x}_{i}-\bar{x}\right)}^{4}/S{D}^{4}-3\end{array}$$

where *n* represents the sample size with a value of *x*_*i*_, $\bar{{x}}$ is the mean value and *SD* is the standard deviation.

A z-test is applied, and z scores can be obtained by dividing the skew values or excess kurtosis by their standard errors^34^:

$$\begin{array}{lll}{Z}_{\gamma 1} & = & \frac{\gamma 1}{S{E}_{\gamma 1}}\\ {Z}_{\gamma 2} & = & \frac{\gamma 2}{S{E}_{\gamma 2}}\end{array}$$

where *SE*_*γ*1_ and *SE*_*γ*2_ are the standard errors of skewness and kurtosis, respectively.

The normality test results with positive skew values and positive excess kurtosis from IBM SPSS software (https://www.ibm.com/analytics/spss-statistics-software) show that the dataset of *TDS* values does not follow the normal distribution (Table 3), as the z score is larger than ± 1.96 (α = 0.05).

**Table 3:** Normality tests of the TDS dataset using skewness and kurtosis.

Parameter	*n*	γ1	*SE*_*γ*1_	*Z*_*γ*1_	γ2	*SE*_*γ*2_	*Z*_*γ*2_
Result	1264	5.34	0.07	77.33	38.98	0.14	282.43

### Outlier detection

The 1264 sets of *TDS* data varied widely (12–2586 mg/L). Tukey’s method^36^ applies to both symmetric and skewed data and detects more outliers for data that do not follow a normal distribution, unlike the standard deviation (*SD*) method (*Mean* ± *2* *SD, Mean ± 3* *SD*)^37^. Since Tukey’s method makes no distributional assumptions about the data^37^, outliers in this study were detected by Tukey’s 3 *IQR* (interquartile range) method. The *IQR* is known as the difference between the first quartile (*Q*1) and the third quartile (*Q*3)^38^:

$$IQR=Q3-Q1.$$

The samples were detected as potential outliers and possible outliers by inner fences with a 1.5 *IQR* interval and outer fences with a 3 *IQR*^37,39^, respectively.

Inner fences are situated at a distance of 1.5 *IQR* below *Q*1 and above *Q*3:

$${\rm{Low}}\;{\rm{potential}}\;{\rm{outliers}}=Q1-1.5\;IQR$$

$${\rm{High}}\;{\rm{potential}}\;{\rm{outliers}}=Q3+1.5\;IQR$$

The intervals with 3 *IQR* are called outer fences and are located below *Q*1 and above *Q*3 at 3 *IQR* distances:

$${\rm{Low}}\;{\rm{possible}}\;{\rm{outliers}}=Q1-3\;IQR$$

$${\rm{High}}\;{\rm{possible}}\;{\rm{outliers}}=Q3+3\;IQR$$

The outlier detection results (Table 4) show that 4.4% and 8.2% of the 1264 *TDS* data account for the possible outliers and potential outliers, respectively.

**Table 4:** Outlier detection results for the *TDS* dataset by Tukey’s 3 *IQR* method.

Parameter	*Q*1	Median	*Q*3	*IQR*	Inner fence	Outer fence
*TDS*, mg/L	74.1	105.6	190.8	116.8	366.0	541.1

### Subbasin selection

The subbasin boundaries used in this study were extracted from the HydroBASINS shapefile^40^, which follows the rule that at every location where two river branches meet, each has an individual upstream area that exceeds a certain size threshold (i.e., 100 km^2^). The rule still allows smaller subbasins to occur, and we selected the 6^th^-level basin for this database according to the data volume and sampling density.

**Fig. 2:**
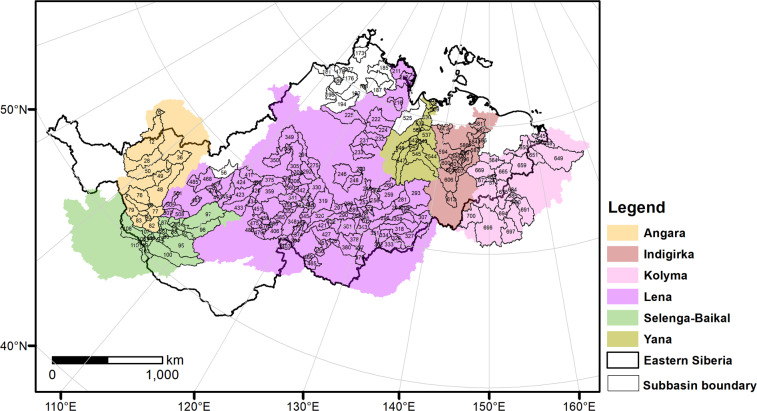
Subbasin location map in eastern Siberia. The six coloured sections represent different river basins; the bold black line corresponds to the eastern Siberian boundary; the thin black line represents the subbasin boundary; the numbers in the figure indicate the subbasin numbers from the HydroBASINS shapefile^40^.

In total, 218 subbasins were selected from a total of 776 subbasins in the eastern Siberia region (including its six major basins) where the sampling sites were located (Fig. 2). Each subbasin was named with a unique code in ObjectID together with average river water chemistry and the climatic factors (temperature (*T*), precipitation (*P*) and potential evaporation (*PET*)) at subbasin scales. *T* and *PET* were derived from the Climate Research Unit (CRU) 4.04 dataset^41^, and *P* was obtained from the Global Precipitation Climatology Centre (GPCC) dataset^42^ at a resolution of 0.5° from 1901 to 2019.

### Meteorological data processing

We clipped the meteorological data (.nc file) using subbasin boundaries and then pre-processed the data to filter out the missing data. The monthly precipitation data (mm/month) of the year are summed to obtain the annual precipitation (mm/year). The same was true for the daily potential evaporation data, which should be multiplied by the number of days of each year. After that, we averaged the meteorological data within each subbasin.

## Data Records

The dataset is publicly available at figshare^43^. The water chemistry database consists of the following 3 categories and associated listed files:

### Category 1: Boundary data

This folder contains the boundaries of eastern Siberia and its six major river basins (Angara, Selenga-Baikal, Lena, Yana, Indigirka and Kolyma) with the river system, which consist of four ***shp*** files.

Eastern_Siberia_boundary.shp

Basin_boundary.shp

Subbasin_boundary.shp

River_system.shp

### Category 2: Water chemistry *data*

This folder contains the full river water chemistry database, which consists of a ***csv*** file with all total dissolved solids (*TDS*) and major ions (Na^+^, K^+^, Ca^2+^, Mg^2+^, Cl^−^, SO_4_^2−^ and HCO_3_^−^), as well as related information (basins, coordinates, sample period, data source, permafrost type, and lithology), basic climatic (temperature and precipitation) and discharge data for each sample (Sample_ID). This folder also contains a sample summary ***csv*** file, which provides the maximum, minimum, mean, standard deviation and number information for the ion concentrations and *TDS* in each river basin.

Samples_database.csv

Samples_summary.csv

### Category 3: Meteorology data

This folder contains the climatic information (temperature, precipitation, and potential evaporation) for the 218 subbasins (named **ObjectID)** on a yearly scale from 1901 to 2019. Each of the 3 files contains a group of 25942 data with 3332 missing values denoted as −9999.

tmp.csv

pre.csv

pet.csv

## Technical Validation

**Fig. 3:**
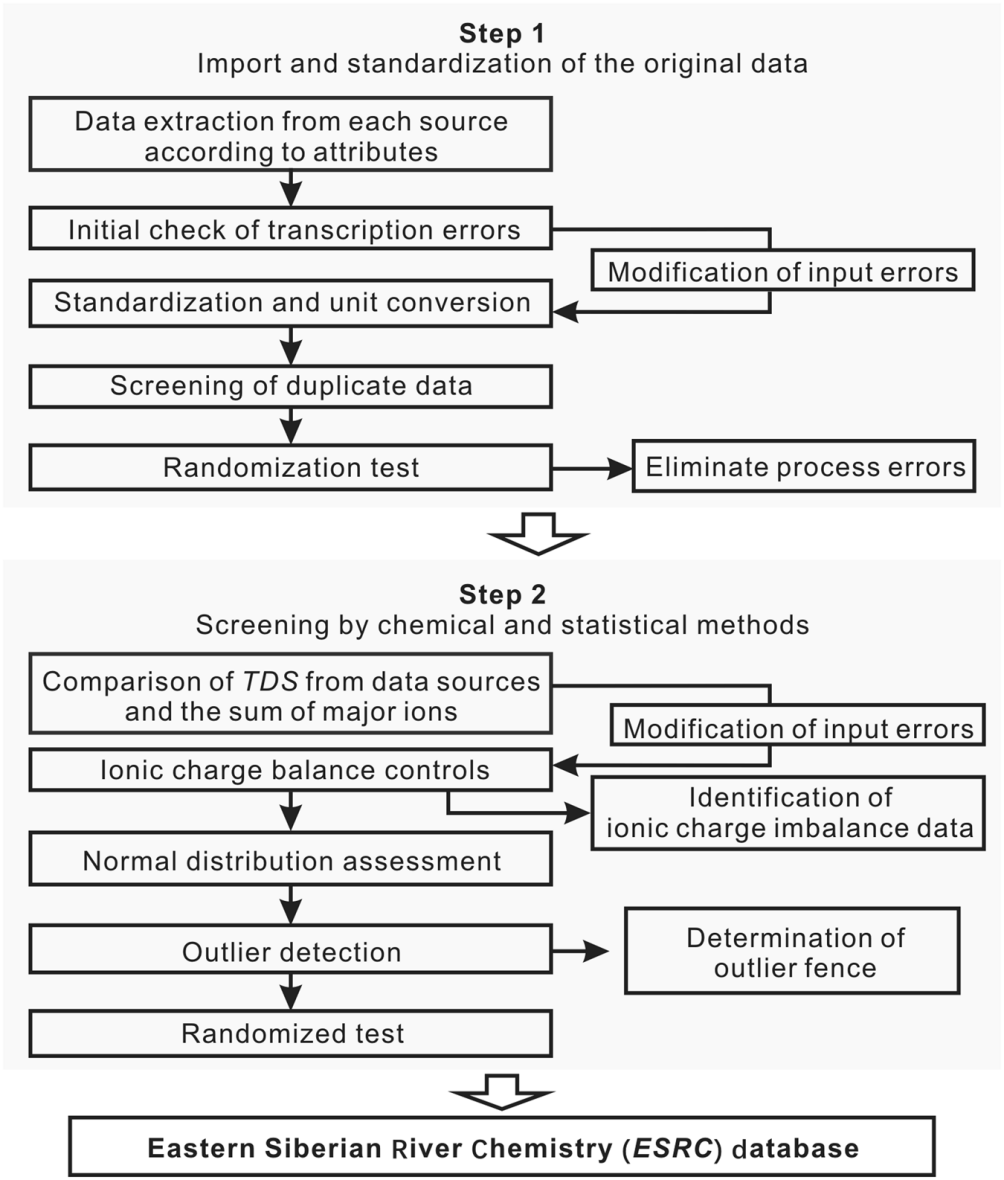
Workflow for Eastern Siberian River Chemistry (***ESRC***) database.

Quality assurance for the 1434 unique datasets from each independent source was separated into two stages (Fig. 3): (1) Import and standardization and (2) Screening by chemical and statistical methods.

### Import and standardization

Data extracted from different sources (manuscripts, online databases and field work reports) were input into an initial data file according to corresponding attributes without alteration. After the multisource data were assembled, an initial check of transcription errors and the modification of input errors (e.g., decimal point mislocation, incorrect placement of variables, and character error) were conducted. Then, standardization and unit conversion were carried out for original water chemical data by parametric conversion (i.e., determining the concentration of hydrogen carbonate by alkalinity and determining the concentration of sulfate by sulfur concentration) and conversion of units into mg/L. Duplicate data were then screened by comparing the coordinates and times of the datasets. Ten percent of random data were selected from our database for validation to eliminate errors during the whole import and standardization process.

### Screening by chemical and statistical methods

We compared the original *TDS* values from data sources (i.e., literature and database) with the calculated *TDS* by the sum of major ions to ensure the rationality of the original ion concentration data. Forty-eight of all datasets were missing ions and marked as “absent” in the “IB” column of the “Samples_database.csv” file. Then, we performed charge balance across all datasets and identified 122 total samples that did not meet the ion balance (marked as “imbalance” in the “IB” column of the “Samples_database.csv” file). The remaining 1264 sets of data were explored by normal distribution assessment and outlier detection methods, and the input and processing of outliers were then verified. Both the inner and outer fences of outliers were determined for the 1264 *TDS* datasets, and outliers with high mineralization of river water appear in only the Angara, Lena and Selenga-Baikal River basins due to different karst processes. Finally, 150 datasets were selected randomly from the final database twice, and the final validation was conducted by people not involved in the data collection process.

### Meteorological data validation

**Fig. 4:**
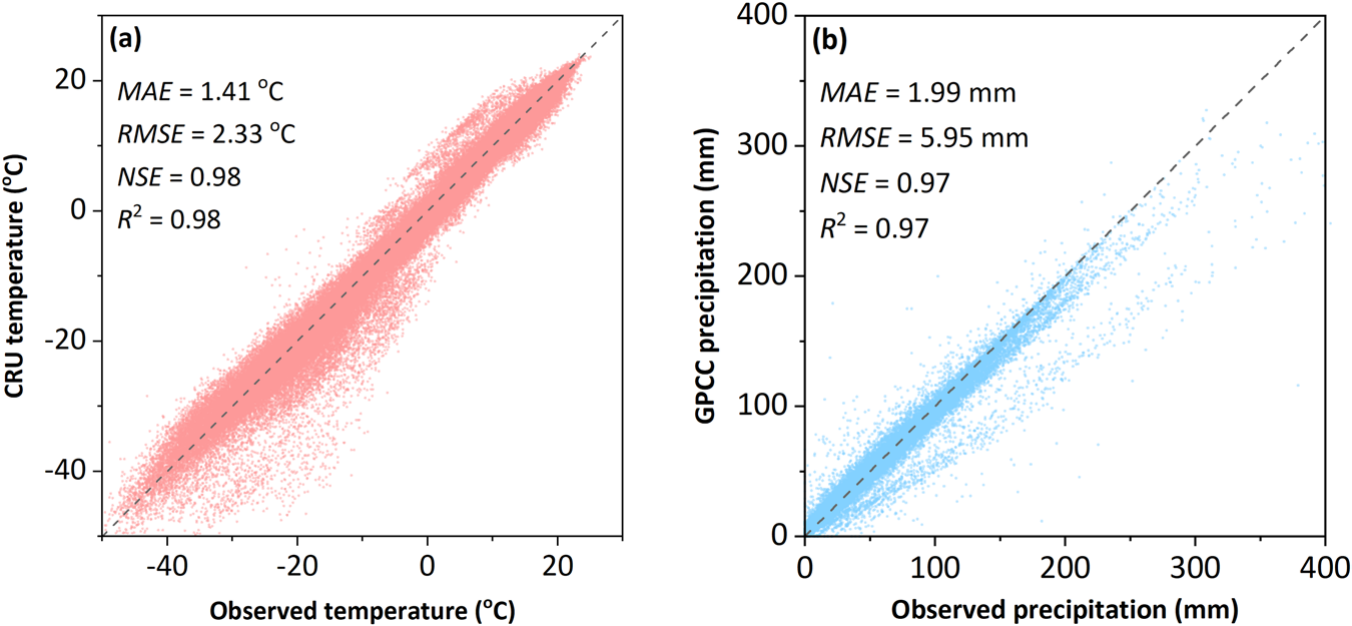
Validation of monthly gridded data against observed data from meteorological stations: (**a**) CRU temperature versus observed temperature; (**b**) GPCC precipitation versus observed precipitation.

To ensure the reliability of the meteorological data, the gridded data were compared with the observation data from the meteorological stations. The validation of monthly gridded data against the observed data (Fig. 4) showed a good performance of CRU temperature products (*MAE* = 1.41 °C, *RMSE* = 2.33 °C, *NSE* = 0.98, *R*^*2*^ = 0.98, *n* = 159889) and GPCC precipitation products (*MAE* = 1.99 mm, *RMSE* = 5.95 mm, *NSE* = 0.97, *R*^*2*^ = 0.97, *n* = 147825).

## Usage Notes

The Eastern Siberian River Chemistry (***ESRC***) database includes the boundaries of eastern Siberia, its six river basins and the 218 subbasins in which water samples were taken. In addition to the sampling information, this database also includes 1434 samples of 7 major ion concentrations, total dissolved solids (*TDS*), climatic factors (temperature and precipitation), lithology, permafrost, sampling information, and annual air temperature, precipitation, potential evaporation, and discharge data for each subbasin during the period from 1901–2019.

### Water chemistry datasets

1. Samples_database.csv

**Sample_ID -** Unique sampling data identifier. The code consists of 2 parts:

- The first part represents the region: SE - Selenga-Baikal; AN - Angara; LE - Lena; YA - Yana; IN - Indigirka; KO - Kolyma; ES - Eastern Siberia.
- The second part represents the sample numbers in each basin.

**Data** - Sampling date in the format YYYY-MM-DD, nondaily sample dates are blank.

**Year -** Sampling years.

**Month -** Sampling month: 1 - January; 2 - February; 3 - March; 4 - April; 5 - May; 6 - June; 7 - July; 8 - August; 9 - September; 10 - October; 11 - November; 12 - December; 7–8 - July to August; 1–12 - annual average data, which do not correspond to a certain month.

**La -** Latitude in unit of decimal degrees.

**Lo -** Longitude in unit of decimal degrees.

**Ca**^2+^**[mg/L] -** Calcium in units of milligrams per litre (mg/L).

**Mg**^2+^**[mg/L]** - Magnesium in units of milligrams per litre (mg/L).

**K**^+^**[mg/L]** - Potassium in units of milligrams per litre (mg/L).

**Na**^**+**^**[mg/L]** - Sodium in units of milligrams per litre (mg/L).

**Cl**^−^**[mg/L]** - Chloride in units of milligrams per litre (mg/L).

**SO**_**4**_^**2**−^**[mg/L]** - Sulfate in units of milligrams per litre (mg/L).

**HCO**_**3**_^−^**[mg/L]** - Hydrogen carbonate in units of milligrams per litre (mg/L).

**TDS[mg/L] -** Total dissolved solids (mg/L) calculated by the sum of seven major ions (Na^+^, K^+^, Ca^2+^, Mg^2+^, Cl^−^, SO_4_^2−^ and HCO_3_^−^).

**pH** - pH value of the sampled water.

**River** - Name of sampled river in English.

**Basin** - Basin to which the sampling location belongs. The basin boundaries are derived from the HydroBASINS shapefile^40^.

**Subbasin** - Subbasin to which the sampling location belongs. The subbasin boundaries are derived from the 6^th^-level HydroBASINS shapefile^40^, and the subbasin code corresponds to the **ObjectID** attribute of Subbasins_boundary.shp in this database.

**T-annual[°C]** - Annual 2-m air temperature (°C) for the corresponding year at the sampling location, which is calculated based on the gridded monthly average 2-m temperature data with a resolution of 0.5° obtained from the Climate Research Unit time series^41^ (CRU TS) v. 4.04.

**T-monthly[°C]** - Monthly average 2-m air temperature (°C) for the month of sampling at the corresponding location, which is sourced from the CRU TS v. 4.04 with 0.5° resolution^41^.

**P-annual[mm]** - Annual precipitation for the corresponding year at the sampling location in units of millimetres (mm), which is calculated based on monthly precipitation data with 0.5° grid resolution prepared by the Global Precipitation Climatology Centre (GPCC)^42^.

**P-monthly[mm]** - Monthly mean precipitation (mm) for the month of sampling at the corresponding location, which is sourced from the GPCC^42^ with a resolution of 0.5°.

**Lithology** - Lithology type of the sampling position based on the new global lithological map database GLiM^44^: **mt** – metamorphic rocks; **pa** - acid plutonic rocks; **pb** - basic plutonic rocks; **sc** - carbonate sedimentary rocks; **sm** - mixed sedimentary rocks; **ss** - siliciclastic sedimentary rocks; **su** - unconsolidated sediments; **va** - acid volcanic rocks; **vb** - basic volcanic rocks; **vi** - intermediate volcanic rocks. Blank means no lithological data are available, and **wb** represents a water body.

**Permafrost type** - 5 permafrost types based on the Northern Hemisphere Permafrost data^45^: **1** - continuous; **2** - discontinuous; **3** - sporadic; **4** - isolated patches; **5** - none. Blank means no permafrost data.

**Citation** - Data source. The numbers correspond to “No.” in Table 1: **1** - ArcticGRO^20^; **2** - GLORICH^21^; **3** - Georgiadi *et al*.^22^; **4** - Huh *et al*.^24^; **5** - Kuzmin *et al*.^23^; **6** - Huh and Edmond, 1999^25^; **7** - Huh *et al*.^26^; **8** - Berkin *et al*.^27^; **9** - Bochkarev, 1959^17^; **10** - Grebenshchikova *et al*.^28^; **11** - Sidorov, 1992^29^; **12** - Shpakova, 1999^18^; **13** - Gabysheva O.I.; **14** - Wang P..

**IB** – Ionic balance results checked by the charge balance method. Samples with absent ions are marked as “absent”; samples with an ion balance (*IB*) greater than 10 are marked as “imbalance”.

**Note** - Remarks during the data acquisition. The HCO_3_ concentrations that were calculated from alkalinity and determined by the charge balance method are marked as “cal_alk” and “cal_ib”, respectively.

**Discharge**[**m**^3^/**s**] - Daily discharge data (m^3^/s).

**Ori_ID** - The original sample ID from the data sources.

**Li**[**mg/L**] - Lithium in units of milligrams per litre (mg/L).

**Sr**[**mg/L**] - Strontium in units of milligrams per litre (mg/L).

**As**[**mg/L**] - Arsenic in units of milligrams per litre (mg/L).

**Ba**[**mg/L**] - Barium in units of milligrams per litre (mg/L).

**Si**[**mg/L**] - Silicon in units of milligrams per litre (mg/L).

^87^**Sr**/^86^**Sr** - ^87^Sr/^86^Sr ratios.

δ ^18^**O-H**_2_**O**[‰] - Oxygen isotope values of water in units of ‰.

δ ^2^**H**-**H**_2_**O**[‰] - Hydrogen isotope values of water in units of ‰.

2) Samples_summary.csv

**Basin** - Basin to which the sampling location belongs. The basin boundaries are derived from the HydroBASINS shapefile^40^.

**Attribute** - water chemistry parameters include 7 major ions and the total dissolved solids in units of milligrams per litre (mg/L): **Ca**^**2+**^ - calcium; **Mg**^**2+**^ - magnesium; **K**^**+**^ - potassium; **Na**^**+**^ - sodium; **Cl**^**–**^chloride; **SO**_**4**_^**2–**^sulfate; **HCO**_**3**_^**–**^hydrogen carbonate; **TDS** - total dissolved solids.

**Statistical variable** - The main statistical results for different hydrochemical parameters in each basin: **Max** - maximum; **Min** - minimum; **Mean** - average value; **Std** - standard deviation; **n** - number of samples.

### Meteorology datasets

The datasets contain 3 files, **tmp.csv, pre.csv** and **pet.csv**, which are air temperature (*T*, °C), precipitation (*P*, mm/yr), and potential evaporation (*PET*, mm/yr) data, respectively. Each file has similar data with two main attributes: **Subbasin ID** (Subbasin) and **yearly data average** (1901 to 2019).

**Subbasin ID** - named by the unique code of the subbasin according **ObjectID**, including a total number of 218 subbasins.

**Yearly data average** - Named by the corresponding year of average annual temperature (precipitation or potential evaporation) within each subbasin from 1901 to 2019. We denote a missing value as −9999.

## Data Availability

Within the repository, we also provide code for extracting climate data of each subbasin from the Climatic Research Unit at the University of East Anglia (http://www.cru.uea.ac.uk/) and the Global Precipitation Climatology Centre (https://climatedataguide.ucar.edu/climate-data/gpcc-global-precipitation-climatology-centre) in the ***code*** folder. ♦ The ***shp*** folder contains 218 subbasin boundary ***shp*** files. ♦ The downloaded input data are stored in 3 ***nc*** files with annual average temperature, annual precipitation, and annual potential evaporation data (1901–2019) in ***yearmean_cru_ts4.04.1901.2019.tmp.dat.nc***, ***yearmean_GPCC_1901–2019_05.nc***, and ***yearmean_cru_ts4.04.1901.2019.pet.dat.nc***, respectively. ♦ The code for extracting data from the .nc file to the .xlsx file was written in Python, and ***extract_tmp-nc_to_xlsx.py***, ***extract_precip-nc_to_xlsx.py***, **and** ***extract_pet-nc_to_xlsx.py*** were used to extract temperature, precipitation, and evaporation data, respectively. ♦ The output data will be stored in .xlsx format (***multi_yr_tmp_subbasins6_1901–2019.xlsx***, ***multi_yr_precip_subbasins6_1901–2019.xlsx***, ***multi_yr_pet_subbasins6_1901–2019.xlsx*** for temperature, precipitation, and potential evaporation data, respectively) in folders ***tmp_clip_sub6***, ***precip_clip_sub6*** and ***PET_clip_sub6*** after running the code.
