# Author Correction: A database of water chemistry in eastern Siberian rivers

**DOI:** 10.1038/s41597-023-01927-4

**Published:** 2023-01-10

**Authors:** Shiqi Liu, Ping Wang, Qiwei Huang, Olga I. Gabysheva, Zehong Li, Jialing Zhang, Ekaterina S. Kazak, Yu Liu, Tcogto Zh. Bazarzhapov, Raisa N. Shpakova, Viktor A. Gabyshev, Sergey P. Pozdniakov, Natalia L. Frolova

**Affiliations:** 1 Key Laboratory of Water Cycle and Related Land Surface Processes, Institute of Geographic Sciences and Natural Resources Research, Chinese Academy of Sciences, 11 A, Datun Road, Chaoyang District, Beijing, 100101 China; 2 University of Chinese Academy of Sciences, Beijing, 100049 China; 3 Institute for Biological Problems of Cryolithozone, Siberian Branch, Russian Academy of Sciences, Yakutsk, 677980 Russia; 4 Department of Hydrogeology, Lomonosov Moscow State University, GSP-1, Leninskie Gory, Moscow, 119899 Russia; 5 School of Environmental Science and Engineering, Guangzhou University, Guangzhou, 510006 China; 6 Baikal Institute of Nature Management of Siberian Branch of the Russian Academy of Sciences, 670047 Ulan-Ude, Russia; 7 Regional Governance and National Policy Department, Moscow State Institute of International Relations, 76, Prospect Vernadskogo, Moscow, 119454 Russia; 8 Department of Land Hydrology, Lomonosov Moscow State University, GSP-1, Leninskie Gory, Moscow, 119991 Russia

**Keywords:** Element cycles, Hydrology, Hydrology, Environmental health, Geochemistry

Correction to: *Scientific Data* 10.1038/s41597-022-01844-y, published online 30 November 2022.

In the Acknowledgements section of this article the grant number relating to the National Natural Science Foundation of China was incorrectly given as E1C10060AE and should have been 42001098.

The original article has been corrected.

